# Targeted Medical Therapy for Vestibular Schwannomas: Evidence, Limits, and Future Directions—A Scoping Review

**DOI:** 10.3390/cimb48030292

**Published:** 2026-03-09

**Authors:** Athena Eliana Arsie, Carlotta Muneretto, Matteo Seno, Marta Gaffeo, Riccardo Nocini, Luca Sacchetto, Silvia Palma, Daniele Monzani

**Affiliations:** 1 Otolaryngology-Head and Neck Surgery Department, University of Verona, 37129 Verona, Italy; athenaeliana.arsie@univr.it (A.E.A.); cm@gmail.com (C.M.); matteo.seno@studenti.univr.it (M.S.); marta.gaffeo@gmail.com (M.G.); riccardo.nocini@univr.it (R.N.); luca.sacchetto@univr.it (L.S.); 2 Audiology, Primary Care Ausl Modena, 41121 Modena, Italy

**Keywords:** sporadic vestibular schwannoma, medical therapy, neurofibromatosis 2 (NF2), bevacizumab, tyrosine kinase inhibitors

## Abstract

Background: Vestibular schwannomas (VSs) are benign tumors that can cause significant morbidity, particularly in neurofibromatosis 2 (NF2) patients, in whom conventional treatments have important limitations. Merlin is a tumor suppressor protein encoded by the Neurofibromin 2 (NF2) gene, and the loss of its function leads to the activation of multiple signaling pathways, providing a rationale for targeted pharmacological therapies. Agents such as bevacizumab and other receptor tyrosine kinase inhibitors (TKIs) have shown variable efficacy but remain limited by toxicity and inconsistent responses. This review aims to evaluate the efficacy and safety of targeted therapies for VSs. Methods: This study was conducted according to PRISMA 2020 guidelines, using a PICO-based search of PubMed, EMBASE, and Scopus to identify studies on pharmacological therapies for VSs published between 2000 and 2025. Eligible human studies included clinical trials and observational studies reporting efficacy, safety, neuroimaging and audiological outcomes. Results: In total, 23 studies were analyzed, predominantly involving NF2-associated VSs. Treatment with bevacizumab was the most frequently investigated medical therapy and yielded the most consistent tumor control along with occasional hearing improvement, albeit with frequent but mostly manageable adverse events. Other targeted agents, including everolimus and TKIs, demonstrated limited or variable efficacy with acceptable toxicity profiles. Conclusions: VSs, particularly in NF2 patients, can cause significant morbidity and are often poorly managed by surgery or radiotherapy. Consequently, targeted medical therapies, especially anti-angiogenic agents, have emerged as valuable alternatives. Bevacizumab shows the most consistent benefits in tumor control, hearing stabilization, and quality of life, despite heterogeneous responses and notable toxicity. Evidence suggests that treatment discontinuation may lead to rapid tumor rebound, highlighting the need for long-term or maintenance strategies and careful monitoring. Future studies are needed to evaluate medical therapy integration with conventional treatments.

## 1. Introduction

Vestibular schwannomas (VSs) are benign, typically slow-growing neoplasms that originate from the Schwann cell sheath of the vestibulocochlear nerve [1,2]. These neoplasms represent the most common benign tumor at the cerebellopontine angle and manifest with symptoms such as hearing loss, tinnitus, vertigo and dizziness. While the majority of VSs occur sporadically, often presenting as unilateral tumors in the fifth or sixth decade of life, approximately 4% to 10% of cases are associated with neurofibromatosis type 2 (NF2-related schwannomatosis) [1,3]. NF2 is an autosomal dominant tumor predisposition syndrome caused by inactivating mutations in the *NF2* tumor suppressor gene on chromosome 22q12 [4]. The *NF2* gene codes for the tumor suppressor Merlin, whose loss of function is the main pathogenic factor in VS pathogenesis.

NF2-associated VSs are typically bilateral, present earlier in life, and exhibit more aggressive growth dynamics [5,6]. These tumors lead to significant morbidity, including progressive sensorineural hearing loss, tinnitus, vertigo, and potentially life-threatening brainstem compression [2,7].

The current standard management for these diseases includes conservative observation (“wait and scan”), microsurgical resection, and stereotactic radiotherapy [4]. However, these modalities have significant limitations, particularly in the NF2 population, among whom surgical resection is associated with a high risk of facial nerve impairment and permanent hearing loss [3,5].

The search for beneficial alternatives has driven recent efforts toward the development of new therapeutic agents based on the principles of targeted therapy. The biological rationale for exploring pharmacological targeted therapies lies mainly in the functional loss of Merlin [8], which acts as a negative regulator of multiple mitogenic signaling pathways. The absence of this regulator leads to the abnormal activation of various receptor tyrosine kinases (RTKs) and downstream cascades, including the PI3K/AKT pathway, the Ras/RAF/MEK/ERK pathway, and the mTORC1 pathway [1,4,9]. Overexpression and activation of the ErbB family, specifically EGFR, ErbB2 and ErbB3, have been consistently observed in VSs, providing concrete molecular targets for therapeutic intervention [1,4]. Other pathways involving vascular endothelial growth factor (VEGF), platelet-derived growth factor receptor (PDGFR), c-Met, and Src have been identified as clinical or preclinical drivers of tumor growth [8,10,11].

Inactivation of NF2 represents the central pathogenic event in both sporadic and NF2-associated VSs, with different underlying mechanisms. In particular, NF2-associated VSs arise from germline or mosaic NF2 mutations, conferring a systemic predisposition to the development of multiple schwannomas and other central nervous system tumors [12].

To date, bevacizumab, a humanized monoclonal antibody targeting VEGF-A, has demonstrated the most significant clinical benefit, such as tumor shrinkage and hearing improvement [6]. Bevacizumab exerts its effect by selectively binding VEGF-A, thereby preventing its interaction with endothelial VEGF receptors and inhibiting tumor-associated angiogenesis [13,14]. VEGF blockade not only limits neovascularization but also reduces vascular hyperpermeability [12].

Beyond anti-angiogenic therapy, several agents targeting signaling pathways deregulated by Merlin loss have been evaluated. Small-molecule tyrosine kinase inhibitors (TKIs) such as lapatinib, icotinib, and erlotinib act by reversibly inhibiting EGFR and/or ErbB2, thereby suppressing downstream signaling cascades including ERK1/2 and AKT [9].

Axitinib is a highly potent oral inhibitor of VEGFR-1, -2, and -3, with additional activity against PDGFRα/β and c-KIT [10]. Given that Schwann cells express PDGF receptors that directly promote their proliferation, axitinib offers a dual mechanism of action by both starving the tumor through VEGFR inhibition and directly suppressing Schwann cell growth via PDGFR blockade [10].

Pazopanib exhibits a similar inhibitory profile, targeting VEGFR, PDGFR, and c-KIT to impair angiogenesis and tumor cell survival [15].

Another therapeutic target in NF2 is represented by the mTOR signaling axis. In this case, Merlin loss leads to constitutive mTORC1 hyperactivation, which acts as a central metabolic switch coordinating protein synthesis and cell cycle progression in response to nutrient and energy availability [9,16]. Everolimus, an oral rapamycin derivative and selective mTORC1 inhibitor, reduces cellular proliferation and may indirectly suppress VEGF production, thereby exerting additional anti-angiogenic effects [9].

Despite these developments, there is a lack of prospective data on maintenance therapy and the optimal dosing regimens needed to balance efficacy with toxicity over time [13,16]. The aim of this review is to synthesize clinical outcomes and to identify the most promising molecular targets and current clinical limitations.

## 2. Materials and Methods

The literature review was conducted in accordance with the criteria outlined in the *Preferred Reporting Items for Systematic Reviews and Meta-Analyses* (PRISMA) 2020 [13]. The review question was formulated using the PICO framework. The population consisted of patients with acoustic neuroma/vestibular schwannoma, irrespective of age, sex, or tumor size. Interventions included systemic or targeted pharmacological therapies, such as anti-VEGF agents (e.g., bevacizumab), mTOR inhibitors (e.g., everolimus, sirolimus), antiangiogenic agents, molecular targeted therapies, and other emerging pharmacological approaches. Comparisons involved no treatment, placebo, surgical resection, radiotherapy, or other conservative treatment modalities. Outcomes assessed included treatment efficacy and safety, such as tumor control through neuroimaging or volumetric regression, hearing preservation, reduction in vestibular symptoms, treatment-related toxicity and adverse events, progression-free survival (PFS), and overall survival (OS).

The literature search was performed using PubMed/MEDLINE, EMBASE, and Scopus. Database-specific search strategies were developed using combinations of controlled terms (MeSH/Emtree) and text words related to vestibular schwannoma, pharmacological therapies (including targeted and antiangiogenic agents), and treatment outcomes. Searches were limited to English-language articles published between 2000 and 2025 in order to ensure the inclusion of literature reflecting contemporary diagnostic criteria and clinical practices, thereby enhancing the relevance and applicability of the findings to current clinical settings. Full electronic search strategies are available in the Supplementary Materials.

Following the initial literature search, the full texts of potentially eligible studies were retrieved and screened for final inclusion. All records identified during the initial search were independently reviewed by three authors (CM, MG, and MS). Any discrepancies were resolved through discussion or, when necessary, by consultation with a senior reviewer (AEA). A second screening phase involved full-text assessment conducted independently by four reviewers (AEA, CM, MG, and MS). Titles and abstracts were screened to identify relevant studies.

Studies were included if they met all the following criteria:

- Publication in the English language;
- Available as full-text articles;
- Studies conducted using human subjects;
- Clinical trials, case series including ≥5 patients, systematic reviews, and prospective or retrospective studies;
- A primary focus on the pharmacological treatment of vestibular schwannoma;
- Reported outcomes related to efficacy, safety, or tumor control, including MRI and/or audiological results.

The exclusion criteria were as follows:

- Single case reports;
- Non-English publications;
- Preclinical studies, including animal or in vitro investigations;
- Studies addressing schwannomas in anatomical locations other than the vestibular nerve or studies on neurofibromatosis without a clear distinction of vestibular schwannoma.

In total, 702 records were initially identified through database searches. After removing duplicates, 486 records remained for assessment. Of these, 69 full-text articles were selected for further evaluation. Full texts were then assessed according to predefined inclusion and exclusion criteria. Discrepancies in the selection process were resolved, when necessary, by consulting a senior reviewer (DM, LS). Ultimately, 22 articles were included. For each study, key information was extracted, including authorship, country of origin, year of publication, sample size, drug, dosage and schedule, adverse events, and main findings.

## 3. Results

A summary of the 22 selected articles is presented in Table 1. Seven studies were prospective, eleven were retrospective, and four were clinical trials, for a total of 685 cases.

All studies primarily included NF2-associated cases, with the exception of one study [24], in which NF2 was an explicit exclusion criterion and the population consisted exclusively of patients with sporadic VSs. This study, in which all 361 patients were treated with aspirin or other non-steroidal anti-inflammatory drugs (NSAIDs), confirmed that aspirin and other NSAIDs do not exert a significant effect on tumor growth. For reasons of methodological transparency and completeness, this study was included in the selection process and reported in the summary table, but it was ultimately excluded from the quantitative and qualitative analysis, as it was not comparable with the remaining cohorts. The final population analysis consisted of 324 NF2 cases. In nine of these studies, only adult patients were included; in one study, only pediatric patients were considered; and in the remaining 12 studies, mixed populations were used.

Based on information reported on previous tumor treatments, 110 subjects had undergone prior surgery, while 23 had received prior radiotherapy. Most studies reported pre-treatment imaging and audiological data, except for one [14], which lacked neuroimaging data. Four studies [6,24,28,30] included a control group.

Bevacizumab was used in 68.2% of the studies (15/22), representing around 76% of the total cases, while everolimus was used in 3 of 22 studies [15,16,17]. Among the remaining studies, two studies [3,9] involved lapatinib and icotinib, one used axitinib [10], and one used erlotinib [29].

### 3.1. Bevacizumab

Detailed schedules of bevacizumab doses are provided in Table 2.

The median follow-up was 25.2 months. Concerning imaging outcomes, assessed by MRI, a 20% change in tumor volume was considered clinically significant, an increase of >20% as disease progression, a decrease of >20% as disease regression, and a decrease of <20% as stable disease [32].

The detailed neuroimaging outcomes from the 15 studies using bevacizumab, encompassing a total of 242 treated patients, are as follows: stable in 141 (58.2%), progression in 17 (7.2%), and progression in 84 (34.6%). Regarding audiological outcomes, data were heterogeneous, with results sometimes reported per patient and other times per ear, often without precise numerosity. Overall, 8 of 15 studies described a deterioration of hearing among a portion of the population, most reported patients with stable hearing, and five studies reported a subset of patients showing hearing improvement. Functional hearing outcomes were evaluated using PTA (Pure Tone Average) and WRS (Word Recognition Score).

Regarding adverse events associated with bevacizumab (Table 3), the studies overall indicate a high frequency of events, predominantly mild to moderate (Grade 1–2), whereas severe events (Grade ≥ 3) are less common but not negligible. In most cases, a permanent discontinuation of therapy was not necessary. Grade 3 events were reported in a minority of patients, while Grade 4–5 events were rare, except for a single death due to cerebral hemorrhage [26]. The Common Terminology Criteria for Adverse Events (CTCAE), developed by the National Cancer Institute (NCI), was used to discriminate adverse effects; it classifies the severity of adverse events (AEs) from Grade 1 to 5 [33].

**Figure 1:**
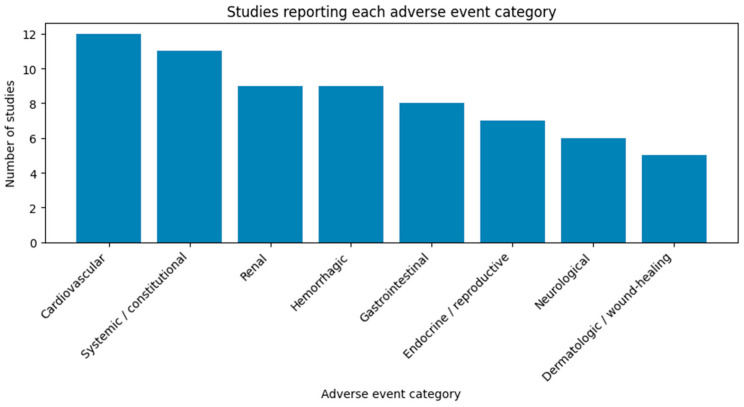
Number of studies reporting each category of adverse events. Each category was counted only once per study, regardless of the number or severity of reported adverse events.

Figure 1 presents the number of studies reporting each specific complication, stratified by the type of adverse event.

### 3.2. Everolimus

Three studies investigated the use of everolimus at a dose of 10 mg/kg/day orally, reducible to 5 mg/kg/day in pediatric patients or in cases of toxicity. In none of these studies was efficacy observed regarding tumor volume reduction or improvement in audiological outcomes. Tumor regrowth and deterioration of hearing function were reported upon drug discontinuation, suggesting the drug’s potential role in preserving hearing and possibly delaying the need for surgery or radiotherapy [16]. Overall, the toxicity profile was predominantly mild to moderate (Grade 1–2). The most frequently reported side effects included mucositis or oral ulcers, fatigue, headache, skin manifestations such as rash or acne, and metabolic disturbances, including hypercholesterolemia and hypertriglyceridemia. Severe adverse events (Grade ≥ 3) were sporadic, including pneumonia, basal cell carcinoma, and pulmonary toxicity, along with a single Grade 4 event of hypertriglyceridemia. No Grade 5 events were observed. Treatment modifications were limited, and permanent discontinuation was necessary in only one patient due to pulmonary toxicity [17]. Overall everolimus appears ineffective at inducing tumor shrinkage. However, the available studies suggest that the drug may contribute to disease stabilization in some patients [9,15], as everolimus appears more cytostatic than cytotoxic [15,16].

### 3.3. Tyrosine Kinase Inhibitors (TKI)

For TKI use, the results are as follows: Plotkin [29] administered erlotinib with a 36-month follow-up and predominantly mild adverse events; no significant clinical responses were observed. Karajannis [9] administered lapatinib to 17 patients, and the 14-month follow-up showed promising but preliminary results, with volumetric tumor reduction in approximately 24% of cases and hearing improvements in 31%. Zhao [3] administered icotinib to 11 patients; the drug was well tolerated and produced positive outcomes. Lastly, Garcia [10] administered axitinib. The treatment was well tolerated but yielded only moderate responses regarding tumor volume and hearing function.

## 4. Discussion

Recent insights into the molecular and cellular mechanisms underlying tumor pathogenesis have enabled the development of biologically targeted therapies for tumors that are otherwise difficult to treat. In particular, recurrent schwannomas following irradiation or previous surgery currently lack effective chemotherapeutic options. Moreover, in patients with NF2, bilateral VSs frequently result in severe/profound hearing loss. Medical treatment is also indicated for patients with progressive tumors in their only hearing ear or in tumor progression when standard options have been exhausted [15,21,34,35].

Pharmacological therapy offers a systemic approach, which is crucial in NF2 patients who often present not only with bilateral VSs but also with meningiomas and ependymomas, which are all potentially responsive to systemic therapy [14]. On the other hand, existing studies are often characterized by small sample sizes and heterogeneous patient populations, complicating the ability to draw definitive clinical conclusions [3,16].

This review confirmed that bevacizumab is currently the most effective pharmacological therapy for the treatment of progressive VSs in NF2 [10,21,28], although additional studies are required to further clarify the drug’s effects [36]. It also remains critical to identify which patient populations may benefit most from this treatment and the optimal overall duration of therapy. During a maintenance regimen, prolonged treatment interruptions (≥3 months) are associated with significant tumor regrowth. Additionally, some patients who had previously achieved a partial response failed to regain the same degree of tumor reduction [28]. Overall, the sources indicate that discontinuing therapy after achieving stability or a positive response is not advisable without extremely close follow-up, suggesting the need for an intensive clinical surveillance lasting at least 6 months following any interruption of anti-VEGF therapy [15,20]. “Rebound growth” is defined as an increase in VS volume exceeding 20% relative to the pre-discontinuation tumor volume, occurring within six months after stopping bevacizumab [21]. Volumetric data underscore that tumor growth following bevacizumab cessation can be extremely rapid and substantial. This phenomenon can be interpreted as a direct consequence of the predominantly suppressive nature of currently available targeted therapies [21]. These agents act by transiently “freezing” or normalizing dysregulated biological processes. Another hypothesis for bevacizumab is attributed to the persistence of a residual vascular scaffold within the tumor. A dormant vascular framework composed of basement membrane structures and pericytes could remain intact during treatment and upon drug withdrawal, enabling rapid vessel reperfusion or neoangiogenic regrowth along these pre-existing vascular “tracks” [2].

During prolonged therapy, increases in circulating hypoxia-induced cytokines and alternative pro-angiogenic factors, such as placental growth factor and hepatocyte growth factor, have also been observed. This accumulation of pro-tumorigenic signals may further amplify tumor regrowth immediately after bevacizumab discontinuation, acting as a molecular “accelerator” once the VEGF blockade is lifted [6].

A similar “released brake” effect was proposed for therapies targeting mTORC1 or EGFR signaling, which are predominantly cytostatic [18]. In the context of persistent Merlin deficiency, these agents impose an artificial block on constitutively active growth pathways. Following treatment cessation, tumor cells that had been arrested in the G1 phase re-enter the cell cycle synchronously, resulting in rapid tumor regrowth [3,16].

A significant emerging factor is the different response observed between adults and children. Children tend to have higher pre-treatment tumor growth rates [6,33] and exhibit significantly lower rates of tumor shrinkage when compared with adults, although children may still derive meaningful benefits in disease stabilization and quality of life.

In the pediatric population, the primary therapeutic goal is therefore not always complete tumor eradication, but rather the long-term preservation of functional hearing and facial nerve integrity [10,35]. The resistance to tumor shrinkage observed in this population can be explained by a convergence of genetic, biological, and microenvironmental factors. Pediatric VSs are more frequently associated with severe clinical phenotypes and are characterized by a higher prevalence of truncating NF2 mutations that result in complete loss of merlin function and drive a markedly more aggressive proliferative program (as reflected by significantly elevated cellular density and proliferation indices). Consequently, these tumors are intrinsically less susceptible to pharmacologically induced volumetric regression [6,12].

Pediatric tumors exhibit a distinct angiogenic profile. Biomarker analyses indicate that, during treatment with bevacizumab, children tend to maintain higher circulating levels of free VEGF [6]. This factor limits the duration and effectiveness of vascular normalization, favoring stabilization rather than true tumor shrinkage. In the context of high baseline proliferative activity, these therapies function mainly as a “brake” on tumor growth rather than inducing meaningful reduction in already established tumor masses [6,35]. Finally, therapeutic response in pediatric patients may be further influenced by prior surgical intervention. Partial resections, commonly performed to decompress the internal auditory canal or preserve neurological function, can alter the tumor microenvironment by inducing inflammatory responses and releasing growth factors such as VEGF and matrix metalloproteinases. These post-surgical changes may stimulate residual tumor tissue and contribute to the greater heterogeneity and reduced predictability of medical treatment outcomes in the pediatric setting [6].

Studies indicate that hearing response is inversely associated with baseline levels of hepatocyte growth factor (HGF), suggesting the possible role of HGF in resistance mechanisms or as a marker of a tumor microenvironment more susceptible to bevacizumab-induced vascular normalization. In contrast, the neuroimaging response is associated with elevated baseline levels of VEGF-D and SDF1α, biomarkers reflecting increased vascular permeability and a strong dependence of the tumor on angiogenic signaling. Overall, these findings support the hypothesis that bevacizumab acts through two distinct mechanisms: a reduction in the intratumoral edema and a reduction in the hyperpermeable vascular component. However, studies employing higher dosing regimens have not consistently confirmed these correlations, suggesting that dose intensification may alter these underlying biological equilibria [14,28].

One notable finding from the literature is the discrepancy between clinical outcomes (as measured by physicians) and patient-reported well-being. Several studies have documented significant improvements in NF2-specific quality of life scores (NFTI-QOL) within 3–6 months of treatment, often independent of neuroimaging or objective hearing responses. Patients report feeling better because therapy alleviates symptoms not easily captured by imaging, such as aural pressure, balance disturbances, and pain [7,29]. Bevacizumab treatment is also associated with reduced tinnitus-related distress in approximately 60% of patients. Studies using instruments such as the Tinnitus Reaction Questionnaire (TRQ) have shown significant decreases in the psychological burden of tinnitus, contributing substantially to the overall perception of benefit [7,21].

Numerous in vitro preclinical studies have investigated innovative molecularly targeted therapeutic strategies for VSs, providing relevant insights for future clinical applications. In particular, Lu et al. identified cellular senescence as a therapeutically exploitable vulnerability in NF2-associated VSs, demonstrating that genotoxic agents induce a stable senescent phenotype mediated by the DNA damage–p21 axis and accompanied by activation of the senescence-associated secretory phenotype (SASP) [37]. Selective elimination of senescent cells using the senolytic agent navitoclax effectively induced apoptosis, supporting a sequential “one–two punch” therapeutic strategy [37]. Other studies have highlighted the efficacy of combined targeted approaches, such as the simultaneous inhibition of BET proteins and FAK, which produced synergistic antitumor effects in NF2-deficient VSs models [38]. In parallel, NF-κB signaling has emerged as a key regulator of tumor proliferation and a promising pharmacological target. Several TKIs, including nilotinib, ponatinib, cabozantinib, and saracatinib, have demonstrated marked antiproliferative effects through the inhibition of PDGFR, c-Met, Src, and downstream PI3K/AKT/mTOR pathways [7,11,38]. Additional approaches include the inhibition of ErbB, JNK, FAK, PI3K/PAK, and mTORC1/2, as well as the use of multitarget agents and nanotherapeutics, all of which have shown the ability to suppress tumor growth in experimental models [39,40,41]. Collectively, these findings reinforce the rationale of developing targeted and combinatorial therapies for VSs and underscore the need for further translational and clinical studies.

## 5. Conclusions

Medical therapy is not intended to replace surgery but rather to complement it as a means of maintaining quality of life and minimizing the iatrogenic harm associated with invasive treatments. Based on the available literature, there is still no formal consensus regarding the optimal dosage, treatment schedule, or duration of therapy. Additionally, the rarity of NF2 renders randomized clinical trials difficult.

Ultimately, clinical trial endpoints should not be limited to tumor volume reduction, as the true goal of treatment is the preservation of neural function and psychological well-being. Patient management should integrate objective measures (MRI/audiometry) with patient-reported outcomes, since reductions in tinnitus-related distress and improvements in quality of life often justify continuing therapy even in the absence of tumor shrinkage.

## Figures and Tables

**Table 1:** Summary of the analyzed articles. Targeted agents evaluated in the included studies: bevacizumab (anti-VEGF monoclonal antibody), everolimus (mTOR inhibitor), lapatinib (dual EGFR/HER2 tyrosine kinase inhibitor), axitinib (VEGFR tyrosine kinase inhibitor), pazopanib (multitarget VEGFR tyrosine kinase inhibitor), erlotinib and icotinib (EGFR tyrosine kinase inhibitors), and aspirin (nonsteroidal anti-inflammatory drug).

Author and Country	Year	Study Design/Sample/ Follow-Up in Months	Drug	Schedule-Dosage	Adverse Events	Findings
Zhao [3]; China	2022	Trial, 11 cases, 12 months	Icotinib	125 mg 3 times daily in 28-day cycles, for up to 12 cycles.	mild (86%): mainly rash, myalgia, and nausea/gastrointestinal pain. No grade 4 toxicities.	Stabilize tumors in some NF2 patients. Occasional hearing improvement.
Plotkin [6]; USA	2019	Prospective, 22 cases, 24 months	Bevacizumab	10 mg/kg of bevacizumab intravenously every 2 weeks for a total of 6 months.	Adverse events occurred in 90.9% of cases, mostly Grade 1–2. Grade 3–4 toxicity in 13.6%.	In NF2, no hearing or tumor response; improved quality of life.
Karajannis [9]; USA	2012	Trial, 17 cases, 14 months	Lapatinib	Adults received 1500 mg once daily; pediatric patients received 900 mg/m^2^ twice daily (maximum 750 mg twice a day).	Toxicity mostly mild (rash, diarrhea, fatigue, liver enzyme elevation); one Grade 3, no Grade 4.	Modest antitumor and hearing benefits in NF2, limited response.
Garcia [10]; USA	2025	Prospective, 12 cases, 12 months	Axitinib	Initial dose of 5 mg twice daily for 4 weeks, with adjustments based on toxicity.	1 grade 3; all other toxicities were grades 1–2, mainly diarrhea, hypertension, hematuria, and fatigue.	Modest tumor and hearing responses in NF2.
Gugel [14]; Germany	2019	Retrospective, 9 cases, 28 months	Bevacizumab	5 mg/kg every 2 weeks, with dose reductions over time to limit long-term toxicity.	5 mild side effects (fatigue, dry skin/mucous membranes) and 2 grade 3 events (proteinuria and hypertension).	modestly slow tumor growth and hearing loss, especially postoperatively.
Hawasli [15]; USA	2013	Retrospective, 6 cases, 9 months	Bevacizumab, Pazopanib	Bevacizumab 5–10 mg/kg/day every 2–3 weeks. Pazopanib 600 mg/day. Treatment duration was from 4 to 21 months, with a mean of 9.1 months.	Only Grade 1 events (anorexia, hypertension, chest pain, epistaxis); Grade 2 events (abdominal pain, diarrhea, weight loss) in 2 patients.	Generally stable disease, hearing mostly maintained, improvement in a few cases.
Goutagny [16]; France	2015	Trial, 10 cases, 24 months	Everolimus	10 mg/day, with dose reduction to 5 mg/day allowed in cases of toxicity.	No severe toxicities. Mild-to-moderate side effects such as mouth ulcers, rash, headache, and fatigue.	Stabilized tumor growth and preserved hearing in NF2; regrowth after stopping the drug.
Plotkin [17]; USA	2023	Prospective, 20 cases, 24 months	Bevacizumab	Induction: 10 mg/kg biweekly (months 1–6); maintenance: 5 mg/kg every 3 weeks (months 7–24).	95% experienced adverse events, with 35% being grade 3; no grade 4–5 events. Most common side effects: hypertension, fatigue, headache, and epistaxis.	Low-dose maintenance bevacizumab is safe and effective for 18 months in NF2, preserving hearing and tumor stability. Better outcomes in adults.
Karajannis [18]; USA	2014	Trial, 9 cases, 12 months	Everolimus	Adults: 10 mg orally once daily; children: 5 mg/m^2^ orally once daily based on body surface area.	Mostly mild (rash, mouth ulcers, fatigue, headache, anemia); two Grade 3 events. No Grade 4 events.	Safe but ineffective in NF2 patients with progressive VSs; no significant tumor volume reduction.
Nghiemphu [19]; USA	2024	Prospective, 12 cases, 12 months	Everolimus	Adults: 10 mg/day; Adolescents (16–17 years): 3 mg/day.	Serious adverse events included pneumonia (grade 3) and elevated triglycerides (grade 4). Common side effects were mouth ulcers, fatigue, skin rashes, and headaches.	Slowed tumor growth and maintained or improved hearing in many patients.
Douwes [20]; The Netherlands	2024	Retrospective, 17 cases, 29.2 months	Bevacizumab	7.5 mg/kg IV every 3 weeks for at least 6 months or until the first adverse event.	The most common adverse events were hypertension, fatigue, and gastrointestinal disorders, mostly mild to moderate.	tumor regression in about 30% of cases; most had stable disease; hearing improvement or maintenance in 40–53%.
Webb [21]; USA	2023	Retrospective, 19 cases, 5.8 months	Bevacizumab	5–10 mg/kg every 2–3 weeks.	13 patients discontinued treatment; 10 stopped bevacizumab due to toxicity rather than tumor progression.	VS growth control; may improve hearing. After discontinuation, rapid tumor regrowth occurred in many patients.
Killeen [22]; USA	2019	Retrospective, 7 cases, 57 months	Bevacizumab	10 mg/kg every 2 weeks for 6 months, then reduced to 5 mg/kg every 3 weeks as maintenance, with a median treatment duration of 33 months.	Mainly mild-to-moderate side effects (fatigue, vomiting, hypertension, epistaxis, proteinuria). One asymptomatic cerebrovascular event on MRI.	Significantly slows growth of VSs in NF2 patients; tumor stability up to 3 years. No improvement in hearing.
Fujii [23]; Japan	2020	Retrospective, 10 cases, 39 months	Bevacizumab	4 intravenous doses of bevacizumab (5 mg/kg) were given every 2 weeks.	5 grade 1 side effects (menstrual delay, nasal bleeding, eye redness, delayed wound healing, nausea, headache, and diarrhea). No severe toxicities.	A ≥20% reduction in 7/17 tumors (41%). Maximum effect within 3–6 months, attenuation by 12 months. Better response in over 25 years.
Marinelli [24]; USA	2018	Retrospective, 361 cases, 37 months	Aspirin or other NSAIDs	186 of 361 patients were taking ≥81 mg aspirin or other NSAIDs at diagnosis.	NA	It does not significantly affect tumor growth in sporadic VSs, nor does it impact linear progression.
Renzi [25]; multicentric pediatric study	2019	Retrospective, 17 cases, 14 months	Bevacizumab	5–10 mg/kg every 2–3 weeks, with a median duration of 1.2 years.	Grade 2 hypertension in 2 cases and grade 2 hypothyroidism and secondary amenorrhea.	Temporary hearing benefits in many NF2 patients, limited radiologic response. Progression after discontinuation.
Alanin [26]; Denmark	2015	Retrospective, 12 cases, 36 months	Bevacizumab	IV at 10 mg/kg every two weeks for 6 months, then a maintenance dose of 15 mg/kg every three weeks.	One death from cerebral hemorrhage; more frequent side effects, including fatigue, proteinuria, menstrual irregularities, and hypertension.	Can reduce tumors and stabilize or improve hearing in progressive NF2; benefits in regard to balance and tinnitus.
Hochart [27]; France	2015	Retrospective, 7 cases, 48 months	Bevacizumab	Bevacizumab IV at 5 mg/kg (4 patients) or 10 mg/kg (3 patients) every 2 weeks, with a median treatment duration of 11.3 months.	5 patients experienced adverse events: hypertension, osteitis, epistaxis, and non-infectious wound complications.	In pediatric NF2 patients, it can slow tumor growth and sometimes improve hearing.
Blakeley [28]; USA	2016	Prospective, 14 cases, 6 months	Bevacizumab	7.5 mg/kg/day IV every 3 weeks, for a total of 16 doses.	124 adverse events occurred: 121 were Grade 1–2, including fatigue, nausea, headache, mild bleeding, elevated liver enzymes, and proteinuria; 3 were Grade 3.	Safe and effective for NF2 patients with progressive VSs.
Plotkin [29]; USA	2010	Prospective, 11 cases, 36 months	Erlotinib	150 mg daily in 28-day cycles, with a median treatment duration of 7.9 months.	Most adverse events were mild (43 cases of Grade 1–2), with only two Grade 3 events. Common side effects included skin rash, hair thinning, and diarrhea.	Modest tumor and hearing stabilization in some NF2 patients; no significant radiographic or hearing responses.
Morris [30]; UK	2016	Prospective, 61 cases, 23 months	Bevacizumab	First 6 months: 5 mg/kg every 2 weeks or 7.5 mg/kg every 3 weeks (n = 14). Maintenance: reduced dosing of 2.5–5 mg/kg every 4 weeks.	50 experienced at least one adverse event, most commonly fatigue, hypertension, nausea and menorrhagia. Grade 3–4 toxicities in 8 patients.	Reduced or stabilized tumor volume, preserved or improved hearing in most NF2. Lower efficacy in younger patients.
Comes [31]; France	2020	Retrospective, 21 cases, 51.5 months	Bevacizumab	Initial phase: 5 mg/kg every 2 weeks for at least 6 months. Maintenance phase: 5 mg/kg every 3 weeks, then every 4 weeks.	Proteinuria in 19% of patients (4/21). Generalized weakness (asthenia) in 19%. Gastrointestinal disorders.	High rates of tumor control. Functional hearing was preserved in most cases.

**Table 2:** Bevacizumab administration details.

Author	Sample Size	Dosage	Timing	Duration
Plotkin [6]	22	10 mg/kg weeks	Every 2 weeks	6 months
Gugel [14]	9	5 mg/kg	Every 2 weeks	
Hawasli [15]	6	5–10 mg/kg	Every 2–3 weeks	9.1 months
Plotkin [17]	20	Induction: 10 mg/kg maintenance: 5 mg/kg	Every 2 weeks Every 3 weeks	6 months From the 7th to the 24th month
Douwes [20]	17	7.5 mg/kg IV	Every 3 weeks	At least 6 months
Webb [21]	19	5–10 mg/kg	Every 2–3 weeks	
Killeen [22]	7	Induction: 10 mg/kg Maintenance: 5 mg/kg	Every 2 weeks Every 3 weeks	6 months Median 33 months
Fuji [23]	10	5 mg/kg	Every 2 weeks	
Renzi [25]	17	5–10 mg/kg	Every 2–3 weeks	1.3 years
Alanin [26]	12	Induction: 10 mg/kg Maintenance: 15 mg/kg	Every 2 weeks Every 3 weeks	6 months
Hochart [27]	7	5 mg/kg (4 patients), 10 mg/kg (3 patients)	Every 2 weeks	11.3 months
Blakeley [28]	14	7.5 mg/kg/day	Every 3 weeks	48 months
Morris [30]	61	Induction: 5 mg/kg or 7.5 mg/kg Maintenance: 2.5–5 mg/kg	Every 2 weeks Every 4 weeks	6 months
Comes [31]	21	Induction: 5 mg/kg Maintenance: 5 mg/kg	Every 2 weeks Every 3 weeks	6 months

**Table 3:** Adverse events associated with bevacizumab across studies.

First Author	Most Frequent Adverse Events	Predominant Grade	Severe Adverse Events Reported
Plotkin [6]	Hypertension, menstrual irregularities, fatigue, epistaxis	Grade 1–2	Grade 3–4 toxicity (13.6%)
Gugel [14]	Fatigue, dry skin/mucous membranes	Grade 1–2	Grade 3
Hawasli [15]	Hypertension, epistaxis, gastrointestinal symptoms	Grade 1–2	None
Plotkin [17]	Hypertension, menstrual irregularities, fatigue, headache, epistaxis	Grade 1–2	Grade 3 events 35%
Douwes [20]	Hypertension, fatigue, gastrointestinal disorders	Grade 1–2	Not reported
Webb [21]	Hypertension, proteinuria, fatigue	Grade 1–2	Grade 3
Killeen [22]	Fatigue, nausea/vomiting, hypertension, epistaxis, proteinuria	Grade 1–2	Cerebrovascular event (grade 4)
Fuji [23]	Menstrual delay, epistaxis, headache, nausea, diarrhea	Grade 1	None
Renzi [25]	Hypertension, hypothyroidism, secondary amenorrhea	Grade 2	None
Alanin [26]	Fatigue, proteinuria, menstrual irregularities, hypertension	Grade 1–2	Fatal cerebral hemorrhage
Hochart [27]	Hypertension, epistaxis, proteinuria, wound-healing complications	Grade 1–2	None
Blakeley [28]	Fatigue, nausea, headache, mild bleeding, proteinuria	Grade 1–2	Grade 3
Morris [30]	Fatigue, hypertension, nausea, menorrhagia	Grade 1–2	Grade 3–4 toxicities
Comes [31]	Proteinuria, asthenia, gastrointestinal disorders	Grade 1–2	None

## Data Availability

No new data were created or analyzed in this study. Data sharing is not applicable to this article.

## Supplementary Materials

The following supporting information can be downloaded at: https://www.mdpi.com/article/10.3390/cimb48030292/s1.
