# Supplementary material for: Targeted Medical Therapy for Vestibular Schwannomas: Evidence, Limits, and Future Directions—A Scoping Review
Source: Curr Issues Mol Biol. 2026 Mar 9;48(3):292. doi: 10.3390/cimb48030292 (PMC13025009; doi:10.3390/cimb48030292)
Supplement: Supplementary file 1 [file cimb-48-00292-s001.zip › cimb-4156971-supplementary.pdf]

Full electronic search strategies:

The search strings used for article selection on Pubmed were: ((“Vestibular Schwannoma”[Mesh] OR “Acoustic Neuroma” OR “Acoustic Schwannoma” OR “Vestibular Nerve Schwannoma” OR “VIII nerve schwannoma”) AND (“Drug Therapy”[Mesh] OR “Pharmacological Treatment” OR “Medical Therapy” OR “Systemic Therapy” OR “Targeted Therapy” OR “Molecular Therapy” OR “Chemotherapy” OR “Bevacizumab” OR “Everolimus” OR “mTOR inhibitors” OR “VEGF inhibitors” OR “Antiangiogenic Agents”) AND (“Treatment Outcome”[Mesh] OR “Tumor Control” OR “Tumor Regression” OR “Hearing Preservation” OR “Hearing Loss” OR “Adverse Effects” OR “Safety” OR “Efficacy”)) AND (“2005”[Date—Publication] : “3000”[Date—Publication]) AND(english[lang]). (“Vestibular Schwannoma”[Mesh] OR “Acoustic Neuroma”[tiab] OR “Acoustic Schwannoma”[tiab] OR “Vestibular Nerve Schwannoma”[tiab] OR “VIII nerve schwannoma”[tiab]) AND (“Drug Therapy”[Mesh] OR “Pharmacological Treatment”[tiab] OR “Medical Therapy”[tiab] OR “Systemic Therapy”[tiab] OR “Targeted Therapy”[tiab] OR “Molecular Therapy”[tiab] OR “Chemotherapy”[tiab] OR “Pharmacotherapy”[tiab] OR “Medical Management”[tiab] OR “Antiangiogenic”[tiab] OR “Antiangiogenic Agents”[Mesh] OR “Bevacizumab”[tiab] OR “Avastin”[tiab] OR “VEGF”[tiab] OR “VEGF Inhibitors”[tiab] OR “Vascular Endothelial Growth Factor”[Mesh] OR “Everolimus”[tiab] OR “Sirolimus”[tiab] OR “mTOR”[tiab] OR “mTOR inhibitors”[tiab] OR “Tyrosine Kinase Inhibitor”[tiab] OR “TKI”[tiab] OR “Imatinib”[tiab] OR “Lapatinib”[tiab] OR “Erlotinib”[tiab] OR “Gefitinib”[tiab] OR “Targeted Agents”[tiab]) AND (“2000”[Date—Publication] : “3000”[Date—Publication]) AND (english[lang]). The search strings used for article selection on EMBASE were: (‘vestibular schwannoma’/exp OR ‘acoustic neuroma’ OR ‘acoustic schwannoma’) AND (‘drug therapy’/exp OR ‘pharmacological treatment’ OR ‘bevacizumab’ OR ‘everolimus’ OR ‘mTOR inhibitor’ OR ‘VEGF inhibitor’ OR ‘antiangiogenic agent’) AND (‘treatment outcome’/exp OR ‘tumor control’ OR ‘hearing preservation’ OR ‘adverse effect’ OR ‘efficacy’) AND ([2005-2025]/py) AND ([english]/lim).(‘vestibular schwannoma’/exp OR ‘acoustic neuroma’:ti,ab OR ‘acoustic schwannoma’:ti,ab) AND (‘drug therapy’/exp OR ‘pharmacological treatment’:ti,ab OR ‘medical therapy’:ti,ab OR ‘systemic therapy’:ti,ab OR ‘targeted therapy’:ti,ab OR ‘molecular therapy’:ti,ab OR ‘chemotherapy’:ti,ab OR ‘pharmacotherapy’:ti,ab OR ‘antiangiogenic’:ti,ab OR ‘bevacizumab’/exp OR ‘everolimus’:ti,ab OR ‘sirolimus’:ti,ab OR ‘mTOR’:ti,ab OR ‘VEGF’:ti,ab OR ‘tyrosine kinase inhibitor’:ti,ab OR ‘TKI’:ti,abOR ‘imatinib’:ti,ab OR ‘lapatinib’:ti,ab OR ‘erlotinib’:ti,ab OR ‘gefitinib’:ti,ab) AND ([2000-2025]/py) AND [english]/lim

The search strings used for article selection on Scopus were: (TITLE-ABS-KEY(“vestibular schwannoma” OR “acoustic neuroma” OR “acoustic schwannoma”) AND TITLE-ABS-KEY(“drug therapy” OR “pharmacological treatment” OR “bevacizumab” OR “everolimus” OR “mTOR inhibitor” OR “VEGF inhibitor” OR “antiangiogenic agent”) AND TITLE-ABS-KEY(“treatment outcome” OR “tumor control” OR “hearing preservation” OR “adverse effect” OR “efficacy”)) AND PUBYEAR > 2004 AND (LIMIT-TO(LANGUAGE, “English”)).
(TITLE-ABS-KEY(“vestibular schwannoma” OR “acoustic neuroma” OR “acoustic schwannoma”) AND TITLE-ABS-KEY(“drug therapy” OR “pharmacological treatment” OR “medical therapy” OR “systemic therapy” OR “targeted therapy” OR “chemotherapy” OR “pharmacotherapy” OR “bevacizumab” OR “everolimus” OR “sirolimus” OR “mTOR” OR “VEGF” OR “antiangiogenic” OR “tyrosine kinase inhibitor” OR “TKI” OR “imatinib” OR “lapatinib” OR “erlotinib” OR “gefitinib”)) AND PUBYEAR > 1999 AND (LIMIT-TO(LANGUAGE, “English”)).
